# Effects of genetic variants on serum parathyroid hormone in hyperparathyroidism and end-stage renal disease patients: A systematic review and meta-analysis

**DOI:** 10.1097/MD.0000000000010834

**Published:** 2018-05-25

**Authors:** Antonela Matana, Marijana Popović, Vesela Torlak, Ante Punda, Maja Barbalić, Tatijana Zemunik

**Affiliations:** a Department of Medical Biology, University of Split, School of Medicine; b Department of Nuclear Medicine, University Hospital Split, Split, Croatia.

**Keywords:** genetic analysis, meta-analysis, parathyroid hormone, single nucleotide polymorphism, systematic review

## Abstract

**Background:**

Parathyroid hormone (PTH) is one of the principal regulators of calcium homeostasis, crucial for normal functioning of the kidneys, bones, heart, and nervous system. Different pathologic conditions can affect serum PTH level resulting in hyperparathyroidism or hypoparathyroidism. Our study assessed the association of previously reported polymorphisms with the level of PTH (expressed in pg/mL) among individuals with different pathologic conditions affecting PTH level.

**Methods:**

We searched Web of Science, MEDLINE, and Scopus to identify relevant articles published up to July 2017. The search yielded 6967 publications of which 44 fulfilled the inclusion criteria. We conducted meta-analyses for calcium-sensing receptor gene (*CaSR*) rs1801725 polymorphism in patients with primary hyperparathyroidism and vitamin D receptor gene (*VDR*) rs1544410 polymorphism in patients with end-stage renal disease (ESRD).

**Results:**

None of the polymorphisms were significantly associated with PTH levels in the overall population. In subgroup analysis by ethnicity for *VDR* rs1544410 gene polymorphism, we found significant differences under dominant model (*SMD*: −0.18 [−0.32, −0.05], *P* < .01) and AA versus GG comparison (*SMD*: −0.29 [−0.52, −0.06], *P* < .01) in Asian patients with ESRD, while nominally significant results (*P* < .05) were observed for AG versus GG and AA versus GG comparisons in European individuals with ESRD.

**Conclusion:**

Scientific evidence of genetic association of serum PTH level among individuals with different pathologic conditions remains deficient and published results provide weak evidence. Further well-conducted studies on larger sample sets designed according to evidence-based principles are warranted to assure clinically applicable findings.

## Abstract 2

Supplemental Digital Content is available in the text

## 1 Introduction

Parathyroid hormone (PTH) is one of the principal regulators of calcium homeostasis. Synthesis and secretion of PTH are triggered by decreased calcium levels in the blood, while PTH inhibition occurs as a result of increased levels of calcium. PTH production represents the most important mechanism for rapid control of ionized calcium in the extracellular fluid (ECF).^[1]^ Proper calcium balance is crucial for normal functioning of the kidneys, bones, heart, and nervous system.^[2]^ Up to 60% of PTH variability is attributed to genetic factors.^[3]^

Different pathological conditions can influence PTH levels. Hyperparathyroidism is the most common pathological condition caused by excessive secretion of PTH and is usually subdivided into levels of clinical relevance as primary, secondary, and tertiary.^[4]^ Primary hyperparathyroidism is the unregulated overproduction of PTH caused by a single adenoma, multiple adenomas or hyperplasia, and rarely, parathyroid carcinoma.^[4]^ Secondary hyperparathyroidism occurs due to chronic kidney disease, vitamin D deficiency, or other causes of low blood calcium, while tertiary hyperparathyroidism is characterized by excessive secretion of PTH after extended secondary (renal) hyperparathyroidism.^[4]^ The less common pathological condition of PTH deficiency is hypoparathyroidism. Two types of hypoparathyroidism are known: primary hypoparathyroidism, which is a state of inadequate PTH activity, and secondary hypoparathyroidism, a physiologic state in which PTH levels are high in response to a primary process that causes hypercalcemia.^[5]^

Vitamin D plays an essential role in the regulation of serum PTH levels and, consequently in calcium homeostasis. Vitamin D deficiency manifests from an increase in PTH, while an overdose of vitamin D results in a decrease in PTH.^[6]^ Vitamin D acts through its metabolite, 1,25(OH)2D3, on the long-term regulation of parathyroid function. 1,25(OH)2D3 may act on the secretion of PTH and its gene regulation and the regulation of transcriptional activity of genes encoding the calcium-sensing receptor (CaSR) and the vitamin D receptor (VDR).^[7,8]^*CaSR* polymorphisms are one of the genetic determinants of extracellular calcium, and a possible predictor of disorders that affect bone and mineral metabolism. The VDR-encoding gene is widely studied to predict differences in bone density^[9]^ and the risk of osteoporosis.^[10]^

Genetic polymorphisms in *CaSR* and *VDR* have been associated with the serum PTH levels in different pathological conditions that are, among other biochemical traits, characterized by an altered PTH level. The functional consequences of these polymorphisms are yet to be discovered. The normal physiological range of PTH is 12 to 65 pg/mL. For primary hyperparathyroidism, the range of PTH level varies from normal in some patients to >65 pg/mL in most patients, whereas for secondary hyperparathyroidism, the range of PTH levels is dependent on the primary process that causes hypercalcemia, but is above 65 pg/mL.^[11,12]^ The highest values of PTH are associated with end-stage renal disease (ESRD), with levels from 2 to 9 times above the upper physiological range.^[13]^ An understanding of the genetic regulation of serum PTH levels could be of valuable clinical importance.

The goal of this study was to perform a systematic review together with meta-analyses of reported genetic loci associated with serum PTH levels in pathological conditions. We provided meta-analyses among individuals suffering from different pathological conditions that influence PTH levels based on previously published candidate gene studies. Such a comprehensive overview has not been provided so far.

## 2 Materials and methods

### 2.1 Publication search

A systematic literature search was undertaken to identify all published studies investigating the association of genetic variants with the level of PTH in patients with different pathological conditions affecting PTH levels. This systematic review was conducted following PRISMA (Preferred Reporting Items for Systematic Reviews and Meta-Analyses) guidelines.^[14]^ We systematically searched 3 bibliographic databases: Web of Science, MEDLINE, and Scopus to identify relevant articles.

Two authors conducted the search and study selection. Discrepancies concerning study selection were resolved through discussion with the last author of this manuscript. The search was performed on July 4, 2017, using a combination of genetic and phenotype words and Medical Subject Headings terms (see S1 Table for the detailed search strategy).

The search yielded a total of 13,924 publications (5657 identified using Ovid Medline, 5600 using Web of Science, and 2667 using Scopus). After removal of duplicates, 6967 publications were assessed. First, 2 authors independently screened all titles and abstracts of articles identified through the search process, which resulted in the identification of 110 potentially relevant articles. Potentially relevant articles were read in full, and 33 of them fulfilled all inclusion criteria. Then, we supplemented the search by scanning the reference list of the relevant articles and previously published related systematic reviews applying the same standardized process, generating an additional set of 20 articles of which 11 were eligible for inclusion. In total, 44 studies fulfilled all inclusion criteria.

### 2.2 Study selection

Studies were included if they were original studies of human individuals, regardless of sample size. To provide a comprehensive insight into the field, studies on individuals with different pathologic conditions related to the disturbance of PTH levels were included but analyzed separately.

All animal and in vitro studies were excluded, as well as case reports, editorials, comments, and review articles. Studies testing for biallelic single-nucleotide polymorphisms or biallelic insertion-deletion marker type were included; otherwise, they were excluded. Further, studies were included if they reported the number of individuals and appropriate descriptive values for PTH levels for every analyzed genotype. Lastly, we limited the review to studies that were written in the English language.

### 2.3 Data collection and data items

Data were extracted by 2 authors independently using the same standardized form, with any disagreements being resolved by consensus. The following data were extracted from each study included in the systematic review: first author's last name, year of publication, country where the study was performed, ethnicity of participants, type of subjects, sample size (number of cases and controls), sample size by genotype (number of cases and controls for every analyzed genotype), mean values and standard deviation for the PTH levels by genotype (for cases and controls), measurement method, and genotype technology. Whenever a report provided standard errors or mean confidence intervals (CIs), but did not provide standard deviations, they were calculated using standard procedures. The units of measurement of PTH levels in the studies included in the meta-analysis were all converted into pg/mL.

### 2.4 Summary measures and synthesis of results

Studies on individuals with different pathologic conditions were analyzed separately due to methodological restraints. The meta-analysis on patients with the same disease was performed for genetic variants that had at least four data sources.

The analysis was performed by multiple pairwise comparisons (e.g., AA vs Aa, AA vs aa, Aa vs aa) and by assuming dominant (AA + Aa vs aa) or recessive (aa + Aa vs AA) genetic models, in order to compare results. Exceptionally, for CaSR rs1801725 polymorphism in individuals with primary hyperparathyroidism, only the recessive genetic model was used because the majority of the included studies grouped carriers of the minor allele (GT+TT) and compared them with the major allele homozygous genotypes (GG).

A pooled standardized mean difference (SMD) and its 95% CI were calculated in a random or fixed effects model, depending on the presence of heterogeneity among studies. If heterogeneity was detected, the random effects model was used. Otherwise, the fixed effects model was applied.

The Cochran Q test was used to assess the heterogeneity, and it was considered significant at *P* < .05. Heterogeneity was quantified with the *I*^2^ metric, which includes values between 0% and 100%, with higher values denoting a greater degree of heterogeneity. *I*^2^ values of 25%, 50%, and 75% were assigned as low, moderate, and high estimates, respectively.

As we tested the genetic association using 5 genetic models, Bonferroni correction was applied. A *P* value of less than .01 (0.05/5) was considered statistically significant.

All analyses were conducted using R, version 3.3.0. The Hardy–Weinberg equilibrium was tested with the HardyWeinberg package, using HWExact and HWChisq functions. Meta-analysis was performed with the meta package, using meta cont, forest, and funnel functions.

We reported our systematic review according to PRISMA guidelines.^[15]^ This study was exempt from review by an ethics committee because it is a meta-analysis of previously published studies.

### 2.5 Risk of bias across studies and quality assessment of primary studies

The Venice criteria for assessing the strength of cumulative evidence in genetic association studies were used.^[16]^ Briefly, credibility for each meta-analysis was graded on 3 levels: the amount of evidence, consistency of replication, and protection from bias categorized as “strong,” “moderate,” or “weak” (grades A, B, C, respectively). Meta-analysis was rated as 1) strong if it received three A grades, 2) moderate if no C grade received, and 3) weak if it received any C grade in any of the 3 criteria. As nonsignificant meta-analyses always received a C for the consistency of replication criterion and all of our results were nonsignificant for overall populations, the replication consistency score was fixed as C. Consequently, all the meta-analyses were rated as weak, and we did not investigate for the amount of evidence and protection from bias domains. Further, possible publication bias was evaluated graphically with the use of funnel plots and statistically by the Egger test for association with *P* < .05.

We created the Confounding-Selection-Information bias score (CSI score) for quality assessment of primary studies (S2 Table) based on the Venice criteria for assessing cumulative epidemiologic evidence in genetic associations, Newcastle–Ottawa case–control, and quality scores in genetic epidemiology.^[16–19]^ The credibility for each study was graded on 3 levels: confounding risk, selection bias risk, and reproducibility, categorized as “strong,” “moderate,” or “weak” (grades A, B, C, respectively).

### 2.6 Additional analysis

A sensitivity analysis was performed by examining the effects of excluding individual studies. We used this analysis to assess the contribution of each study to the final weighted effect in the analysis. When applicable, subgroup analysis based on ethnicity was performed to assess the potential impact of the source of heterogeneity.

## 3 Results

**Figure 1:**
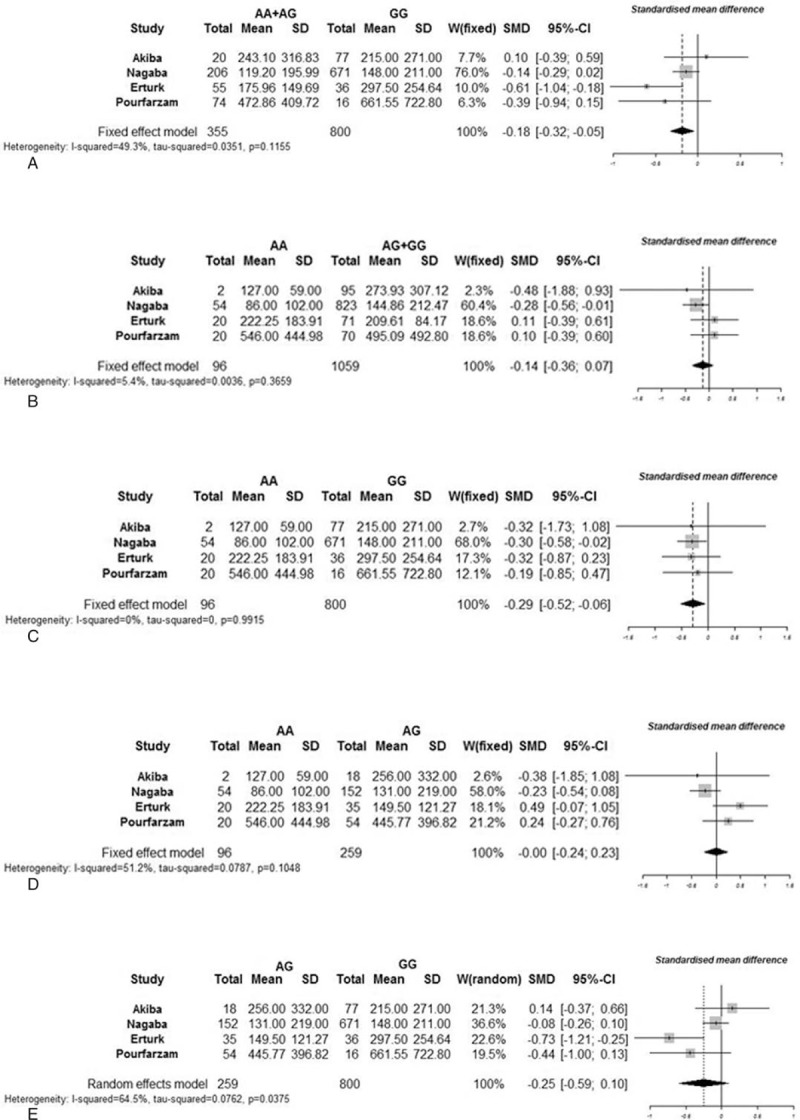
Forest plots for associations between VDR rs1544410 gene polymorphism and PTH level among end-stage renal disease of Asian patients under (A) dominant; (B) recessive; (C) AA versus GG; (D) AA versus AG; and (E) AG versus GG genetic models.

The detailed steps of our literature search are shown in Fig. 1. Forty-four studies performed on individuals with different pathological conditions were included. Included studies provided data regarding 40 distinct polymorphisms in or near 15 different genes. The most widely studied genes were the *VDR* (19 studies) and *CaSR* (10 studies). The majority of the studies were conducted in patients with primary hyperparathyroidism (21%), secondary hyperparathyroidism (18%), or ESRD (14%).

A meta-analysis was possible for *VDR* rs1544410 gene polymorphism in patients with ESRD and *CaSR* rs1801725 gene polymorphism in patients with primary hyperparathyroidism. We did not conduct a meta-analysis for other polymorphisms because fewer than 4 data sources were available for patients with the same disease.

Eight studies^[20–27]^ were included in the meta-analysis for the relationship between *VDR* rs1544410 polymorphism and the PTH level among patients with ESRD. The pooled population comprised 1560 individuals. Three studies were conducted in Europe, 4 in Asia, and 1 in Africa. Table 1 summarizes the characteristics of the included studies. There was no significant result observed for any of the genetic models (Table 2, S1 Figure). No publication bias was detected (*P* = .41, .31, .24, .36, and .50 for dominant, recessive, BB vs bb, BB vs Bb, and Bb vs bb models, respectively; S2 Figure).

**Table 1:**
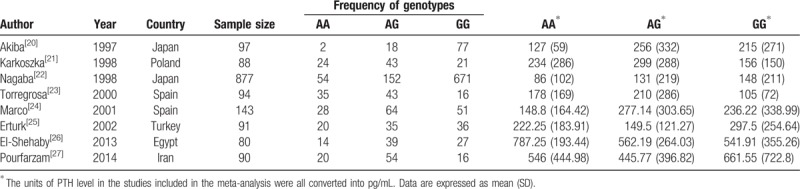
Characteristics of studies included in the meta-analysis for the relationship between VDR *rs1544410* gene polymorphism and PTH level among patients with the end-stage renal disease.

**Table 2:**
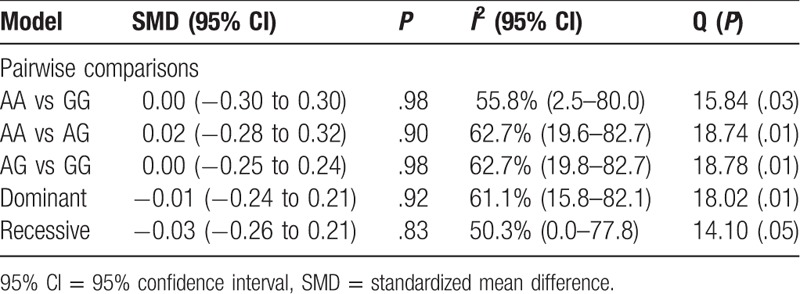
Results of meta-analysis for the association between VDR *rs1544410* gene polymorphism and PTH level among patients with the end-stage renal disease.

Heterogeneity among studies was detected (Table 2); therefore, to ascertain an effect due to ethnic differences, we performed a subgroup analysis based on ethnicity with 3 studies on Europeans and 4 studies on Asians. Marginally significant differences (*P* < .05) among European individuals were observed for AG versus GG (*SMD*: −0.30 [−0.03, −0.57], *P* < .03) and AA versus AG comparisons (*SMD*: −0.28 [−0.55, −0.01], *P* < .04). Heterogeneity was no longer significant for any of the genetic models. Results for the Asian population showed significant differences under a dominant model (*SMD*: −0.18 [−0.32, −0.05], *P* < .01) and AA versus GG comparisons (*SMD*: −0.29 [−0.52, −0.06], *P* < .01) (Fig. 1). High heterogeneity was observed only for the AG versus GG comparison (S2 Figure).

Four studies^[28–31]^ providing 5 data points were included in the meta-analysis for *CaSR* rs1801725 gene polymorphism in patients with primary hyperparathyroidism. The pooled population comprised 308 individuals and all studies were conducted in Europe. Characteristics of the included studies are presented in Table 3.

**Table 3:**
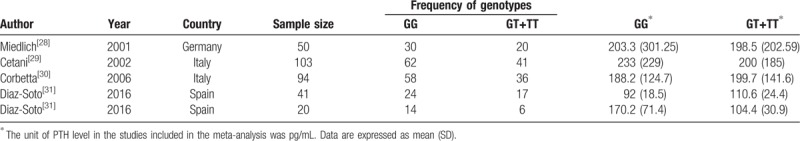
Characteristics of studies included in the meta-analysis for the relationship between CaSR *rs1801725* gene polymorphism and PTH level in primary hyperparathyroidism patients.

No significant result was observed for the recessive model (*SMD*: −0.03 [−0.44, −0.39], *P*-value: .91) (S3 Figure), but a moderate degree of heterogeneity among studies was revealed (*I*^*2*^: 64.2% [5.9%, 86.4%]; *Q* = 11.18, *P*-value: .03). After excluding the study by Diaz-Soto et al^[31]^ from the analysis, the heterogeneity was no longer significant (*I*^*2*^: 0.0% [0.0%, 69.5%]; *Q* = 0.68, *P*-value: .71 while the association remained not significant (*SMD*: −0.04 [−0.22, 0.29], *P*-value < .79). A small sample size could be the reason for the influential role of this study in the heterogeneity among studies. No publication preference was identified (*P* = .85, S4 Figure).

The sensitivity analysis for all meta-analyses revealed that our results were statistically stable, as none of the studies contributed to a lack of a difference in the significance between the estimates.

## 4 Discussion

Our systematic review and meta-analysis is the first comprehensive study to date of published literature regarding polymorphic variants and the level of PTH in a population with pathological conditions affecting PTH serum levels. We found no significant association between reported polymorphisms and PTH levels in patients with primary hyperparathyroidism. The association between *VDR* rs1544410 polymorphisms and PTH levels in patients with ESRD was significant only in minor allele homozygous Asians who had higher PTH levels than patients with the major allele homozygous genotype.

The most frequently investigated polymorphisms in previous candidate studies were those involving the *VDR* and *CaSR* genes. The VDR binds the active form of vitamin D, thus modulating many biological activities of the endocrine, immune, and nervous systems.^[32,33]^*VDR* polymorphisms were widely studied in association with primary hyperparathyroidism,^[34,35]^ secondary hyperparathyroidism in hemodialysis patients,^[22,24,25,27]^ nephrolithiasis,^[36]^ renal failure,^[37]^ sarcoidosis,^[38]^ multiple sclerosis,^[39,40]^ diabetes mellitus type 1,^[41,42]^ diabetes mellitus type 2,^[43,44]^ and osteoporosis.^[45,46]^ Th most commonly studied polymorphisms were rs2228570 (FokI), rs1544410 (BsmI), rs731236 (TaqI), and rs7975232 (ApaI) and, although heavily studied, only a few studies showed associations with genetic loci in different pathological conditions. Lower VDR and higher PTH mRNA levels were associated with primary hyperparathyroidism for minor allele homozygotes of rs1544410 and rs7975232, and major allele homozygotes of rs731236 polymorphism.^[34]^ The level of PTH has been associated with renal failure for rs2228570 major allele homozygotes.^[37]^

We conducted a meta-analysis for rs1544410 *VDR* polymorphism in 1560 patients with ESRD. Recently, 1 meta-analysis on the association of VDR rs1544410 (BsmI) gene polymorphism and serum PTH level was conducted among the 596 patients with ESRD.^[47]^ Results of that meta-analysis revealed that the PTH level in patients carrying the AG genotype was higher than in patients carrying the GG genotype, both in the overall population as well as in Caucasians.^[47]^ Only 1 study on an Asian population was included in the previously mentioned meta-analysis; therefore, the results of the meta-analysis on Asians were not robust.

PTH levels in our study were higher in ESRD patients carrying the GG genotype than in ESRD patients carrying the AA genotype in Asians. Furthermore, we found no association in the overall population, while for Europeans a nominal significance was observed for the AG genotype compared with the GG genotype. Likewise, we found a minimal significance for the AA genotype compared with the AG genotype, which is partly consistent with the previous meta-analysis by Zhang et al.^[47]^ However, our meta-analysis had a larger sample size than the study by Zhang et al,^[47]^ allowing greater statistical power for detecting true associations. As the geographical origin has an effect on genotypic variation among individuals,^[48]^ an additional contribution of our study is the meta-analysis performed on Asian individuals, because no such analysis was carried out in the previously mentioned study.

The *CaSR* plays a major role in the maintenance of the concentration of ionized calcium in the serum through the regulation of PTH circulating levels.^[49]^ Polymorphisms in the *CaSR* gene are considered for association with primary hyperparathyroidism,^[28–31]^ familial hypocalciuric hypercalcemia (FHH) and severe neonatal hyperparathyroidism (NSHPT),^[50,51]^ ESRD,^[52,53]^ and colorectal cancer.^[54–56]^ The most commonly studied polymorphisms of the CaSR are rs1801725 (A986S), rs1042636 (R990G), and rs1801726 (Q1011E). These polymorphisms were studied in patients with primary hyperparathyroidism, but no overall association was found,^[28–30]^ although 1 study showed a correlation between rs1801725 polymorphism and a higher PTH level in normocalcemic hyperparathyroidism patients.^[31]^ The expression of the CaSR was found to be higher in patients with ESRD carrying the rs1042636 and 1801726 homozygous genotypes for the minor allele,^[53]^ while the rs1042636 major allele homozygous genotypes seemed to be associated with the responsiveness of the parathyroid gland to changes in extracellular Ca^2+52^.

Our meta-analysis results showed no association between *CaSR* rs1801725 (A986S) polymorphism (A986S) and PTH level in patients with primary hyperparathyroidism.

The current systematic review and meta-analysis is the first to provide a critical overview of previously reported genetic associations with serum PTH levels in patients with primary hyperparathyroidism as well as a novel and improved meta-analysis in patients with secondary hyperparathyroidism caused by ESRD. Our study revealed several limitations, and therefore, the results must be cautiously considered. The existence of unpublished data and the inability to obtain the raw data from some authors may be a possible source of bias.

Furthermore, although we performed a very extensive and comprehensive literature search, it is possible that some relevant manuscripts have been missed. Our literature search was restricted to papers published in English, so there is a possibility for the systematic exclusion of studies published in other languages. Also, we were unable to adjust our analysis for covariates, such as age and sex. As tests for the assessment of publication bias are underpowered for meta-analyses of 10 or fewer studies, misleading inferences about publication bias could be generated.^[57]^ Another limitation of the study is the use of the invalidated CSI score for quality assessment of primary studies. In addition, almost all studies had at least one C grade for the quality assessment (S3 Table), pointing out drawbacks in study designs, which produces weak evidence. Despite the aforementioned limitations, we intended to provide an original, comprehensive summary of the literature and make conclusions that could be useful in clinical practice.

## 5 Conclusion

This systematic review indicates that the deficiency of genetic association studies on PTH levels among different pathological conditions affected PTH level. Our findings point to the weakness of results of candidate studies published so far and indicate that future well-conducted genome-wide association studies of serum PTH levels among different pathological conditions affecting PTH levels are highly warranted. Studies using a larger sample set and designed according to evidence-based principles would assure clinically important findings that can be used in personalized health care and the treatment of patients.

## Acknowledgments

We would like to thank Ana Utrobičić (Central Medical Library, University of Split, School of Medicine, Split, Croatia) for technical support and Shelly Pranić, PhD (University of Split, School of Medicine, Split, Croatia) for proofreading of the manuscript.

## Author contributions

M.B., V.T., and A.P. participated in the coordination and conception of the study; A.M., M.P., and T.Z. developed and critically assessed the search strategy; A.M. and M.P. performed the search and extracted the data; A.M. performed the data analysis; A.M. and M.P. drafted the manuscript; T.Z. coordinated and conceived the original study idea; T.Z. and M.B. reviewed the manuscript. All authors approved the final version of the manuscript.

**Conceptualization:** Tatijana Zemunik.

**Formal analysis:** Antonela Matana.

**Funding acquisition:** Tatijana Zemunik.

**Investigation:** Antonela Matana, Marijana Popović.

**Methodology:** Antonela Matana, Marijana Popović.

**Software:** Antonela Matana.

**Supervision:** Vesela Torlak, Ante Punda, Maja Barbalić, Tatijana Zemunik.

**Writing – original draft:** Antonela Matana, Marijana Popović.

**Writing – review & editing:** Maja Barbalić, Tatijana Zemunik.

## Supplementary Material

Supplemental Digital Content
