# Supplementary material for: Effects of genetic variants on serum parathyroid hormone in hyperparathyroidism and end-stage renal disease patients: A systematic review and meta-analysis
Source: Medicine (Baltimore). 2018 May 25;97(21):e10834. doi: 10.1097/MD.0000000000010834 (PMC6392899; doi:10.1097/MD.0000000000010834)
Supplement: Supplemental Digital Content [file medi-97-e10834-s001.doc]

**S1 Figure**. Forest plots for associations between VDR rs1544410 gene polymorphism and PTH level among end-stage renal disease patients under A) dominant; B) recessive; C) AA vs. GG; D) AA vs. AG and E) AG vs. GG genetic models.

**S2 Figure**. Funnel plots for VDR rs1544410 gene polymorphism in end-stage renal disease patients under A) dominant; B) recessive; C) AA vs. GG; D) AA vs. AG and E) AG vs. GG genetic models.

**S3 Figure**. Forest plot for association between CaSR rs1801725 gene polymorphism and PTH level in patients with primary hyperparathyroidism under a recessive model.

**S4 Figure**. Funnel plot for CaSR rs1801725 gene polymorphism in primary hyperparathyroidism patients under a recessive model.

**Supplementary information for manuscript:**

**Genetic variants effects on serum parathyroid hormone in hyperparathyroidism and end-stage renal disease patients: a systematic review and meta-analysis**

Antonela Matana, Marijana Popović, Vesela Torlak, Ante Punda, Maja Barbalić, Tatijana Zemunik

**S1 Table. Search strategy.**

| **OvidMEDLINE** |
| --- |
| 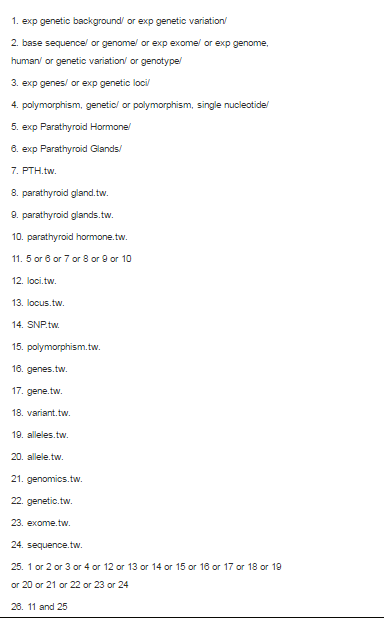 |
| **Web of Science** |
| (TS = (genetic background OR variant OR sequence OR genome OR exome OR genotype OR loci OR polymorphism OR locus OR SNP OR genes OR gene OR variant OR allele OR genomics OR genetic) AND TS=(parathyroid gland OR parathyroid glands OR parathyroid hormone OR PTH)) *AND* LANGUAGE**:** (English) |
| **SCOPUS** |
| TITLE-ABS-KEY ( genetic  background  OR  variant  OR  sequence  OR  genome  OR  exome  OR  genotype  OR  loci  OR  polymorphism  OR  locus  OR  snp  OR  genes  OR  gene  OR  variant  OR  allele  OR  genomics  OR  genetic )  AND  TITLE-ABS-KEY ( parathyroid gland  OR  parathyroid  glands  OR  parathyroid  hormone  OR  pth ) |

S2 Table. Quality assessment for primary studies: Confounding-Selection-Information bias score.

|  | level A grade | level B grade | level C grade |
| --- | --- | --- | --- |
| Confounding risk | 1. No visible confounding (or proper adjustment for possible confounding) 2. No indication of population stratification | 1. Possibility/probability of certain level of confounding 2. Possibility/probability of population stratification | 1. Detectable confounding 2. Strong indication of stratification |
| Protection from bias | 1. Representatives of exposed cohort/case/controls 2. Cohorts/controls in Hardy-Weinberg equilibrium (HWE) | 1. Insufficiently characterized cases or cohorts, control selected from structured sampling frame (hospital, clinic of health care programed based) 2. Cohorts/controls in HWE | 1. No information on cohort/control selection 2. Cohort/controls failed HWE or no information for HWE |
| Reproducibility | NGS or favorable genotyping quality control estimate given with two different genotyping methods, subset or total dataset replicated | Partial genotyping quality control results | No indication of genotyping reproducibility |

S3 Table. Quality assessment of primary studies performed on individuals with different pathological conditions

|  |  | Confounding risk | Protection from bias | Reproducibility |
| --- | --- | --- | --- | --- |
| 1. | Akiba, T., R. Ando, et al. Is the bone mass of hemodialysis patients genetically determined? Kidney Int Suppl 1997;62: S69-71. | B | B | C |
| 2. | Karkoszka, H., J. Chudek, et al. Does the vitamin D receptor genotype predict bone mineral loss in haemodialysed patients? Nephrol Dial Transplant 1998;13(8): 2077-2080. | B | C | C |
| 3. | Nagaba, Y., M. Heishi, et al. Vitamin D receptor gene polymorphisms affect secondary hyperparathyroidism in hemodialyzed patients. American Journal of Kidney Diseases 1998;32(3): 464-469. | B | B | C |
| 4. | Tagliabue, J., M. Farina, et al. BsmI polymorphism of the vitamin D receptor gene in hyperparathyroid or hypoparathyroid dialysis patients. American Journal of Clinical Pathology 1999;112(3): 366-370. | B | B | C |
| 5. | Niimi T, Tomita H, Sato S, Akita K, Maeda H, Kawaguchi H, et al. Vitamin D receptor gene polymorphism and calcium metabolism in sarcoidosis patients. Sarcoidosis Vasculitis and Diffuse Lung Diseases. 2000;17(3):266-9. | B | C | C |
| 6. | Pacheco D, Menarguez J, Cristobal E, Arribas B, Alcazar JA, Carrion R, et al. BsmI vitamin D receptor polymorphism and pathogenesis of parathyroid adenoma. Medical science monitor : international medical journal of experimental and clinical research. 2000;6(4):658-60. Epub 2001/02/24. | B | C | C |
| 7. | Torregrosa JV, Ybarra J, Moreno A, Pons F, Oppenheimer F, Torres A. Vitamin D receptor gene polymorphisms and bone mineral density in patients on hemodialysis. Nephron. 2000;84(4):381-2. Epub 2000/04/08. | B | C | C |
| 8. | Correa P, Lundgren E, Rastad J, Åkerström G, Westin G, Carling T. The NciI polymorphism in the cyclin D1 gene and sporadic primary hyperparathyroidism. Journal of Internal Medicine. 2001;250(6):516-20. | B | C | C |
| 9. | Marco MP, Craver L, Betriu A, Fibla J, Fernandez E. Influence of vitamin D receptor gene polymorphisms on mortality risk in hemodialysis patients. American journal of kidney diseases : the official journal of the National Kidney Foundation. 2001;38(5):965-74. Epub 2001/10/31. | B | A | C |
| 10. | Miedlich S, Lamesch P, Mueller A, Paschke R. Frequency of the calcium-sensing receptor variant A986S in patients with primary hyperparathyroidism. European Journal of Endocrinology. 2001;145(4):421-7. | B | B | C |
| 11. | Yamauchi M, Sugimoto T, Yamaguchi T, Yano S, Kanzawa M, Kobayashi A, et al. Association of polymorphic alleles of the calcium-sensing receptor gene with the clinical severity of primary hyperparathyroidism. Clinical Endocrinology. 2001;55(3):373-9. | B | C | C |
| 12. | Cetani F, Borsari S, Vignali E, Pardi E, Picone A, Cianferotti L, et al. Calcium-sensing receptor gene polymorphisms in primary hyperparathyroidism. Journal of Endocrinological Investigation. 2002;25(7):614-9. | B | C | C |
| 13. | Erturk S, Kutlay S, Karabulut HG, Keven K, Nergizoglu G, Ates K, et al. The impact of vitamin D receptor genotype on the management of anemia in hemodialysis patients. American journal of kidney diseases : the official journal of the National Kidney Foundation. 2002;40(4):816-23. Epub 2002/09/27. | B | B | C |
| 14. | Giannini S, D'Angelo A, Nobile M, Carraro G, Rigotti P, Silva-Netto F, et al. The effects of vitamin D receptor polymorphism on secondary hyperparathyroidism and bone density after renal transplantation. Journal of Bone and Mineral Research. 2002;17(10):1768-73. | C | C | C |
| 15. | Gohda T, Shou I, Fukui M, Funabiki K, Horikoshi S, Shirato I, et al. Parathyroid hormone gene polymorphism and secondary hyperparathyroidism in hemodialysis patients. American Journal of Kidney Diseases. 2002;39(6):1255-60. | B | C | C |
| 16. | Yokoyama K, Shigematsu T, Tsukada T, Hara S, Yamada A, Kawaguchi Y, et al. Calcium-sensing receptor gene polymorphism affects the parathyroid response to moderate hypercalcemic suppression in patients with end-stage renal disease. Clinical Nephrology. 2002;57(2):131-5. | C | C | C |
| 17. | Goertz B, Fassbender WJ, Williams JC, Marzeion AM, Bretzel RG, Stracke H, et al. Vitamin D receptor genotypes are not associated with rheumatoid arthritis or biochemical parameters of bone turnover in German RA patients. Clinical and Experimental Rheumatology. 2003;21(3):333-9. | B | C | C |
| 18. | Ozkaya O, Soylemezoglu O, Misirlioglu M, Gonen S, Buyan N, Hasanoglu E. Polymorphisms in the vitamin D receptor gene and the risk of calcium nephrolithiasis in children. European Urology. 2003;44(1):150-4. | B | C | C |
| 19. | Ak DG, Kahraman H, Dursun E, Duman BS, Erensoy N, Alagol F, et al. Polymorphisms at the ligand binding site of the vitamin D receptor gene and osteomalacia. Disease Markers. 2005;21(4):191-7. | C | C | C |
| 20. | Gago EV, Cadarso-Suarez C, Perez-Fernandez R, Burgos RR, Mugica JD, Iglesias CS. Association between vitamin D receptor Fokl polymorphism and serum parathyroid hormone level in patients with chronic renal failure. Journal of Endocrinological Investigation. 2005;28(2):117-21. | B | B | C |
| 21. | Rubello D, Giannini S, D'Angelo A, Nobile M, Carraio G, Rigotti P, et al. Secondary hyperparathyroidism is associated with vitamin D receptor polymorphism and bone density after renal transplantation. Biomedicine & Pharmacotherapy. 2005;59(7):402-7. | C | C | C |
| 22. | Corbetta S, Eller-Vainicher C, Filopanti M, Saeli P, Vezzoli G, Arcidiacono T, et al. R990G polymorphism of the calcium-sensing receptor and renal calcium excretion in patients with primary hyperparathyroidism. European Journal of Endocrinology. 2006;155(5):687-92. | B | B | C |
| 23. | Kim JG, Kim JH, Kim JY, Ku SY, Jee BC, Suh CS, et al. Association between osteoprotegerin (OPG), receptor activator of nuclear factor-kappa B (RANK), and RANK ligand (RANKL) gene polymorphisms and circulating OPG, soluble RANKL levels, and bone mineral density in Korean postmenopausal women. Menopause-the Journal of the North American Menopause Society. 2007;14(5):913-8. | B | B | C |
| 24. | Vezzoli G, Terranegra A, Arcidiacono T, Biasion R, Coviello D, Syren ML, et al. R990G polymorphism of calcium-sensing receptor does produce a gain-of-function and predispose to primary hypercalciuria. Kidney international. 2007;71(11):1155-62. Epub 2007/03/03. | B | B | C |
| 25. | Eren PA, Turan K, Berber I, Canbakan M, Kara M, Tellioglu G, et al. The clinical significance of parathyroid tissue calcium sensing receptor gene polymorphisms and expression levels in end-stage renal disease patients. Clinical Nephrology. 2009;72(2):114-21. | B | B | C |
| 26. | Giannini, S., S. Sella, et al. Persistent Secondary Hyperparathyroidism and Vertebral Fractures in Kidney Transplantation: Role of Calcium-Sensing Receptor Polymorphisms and Vitamin D Deficiency. Journal of Bone and Mineral Research. 2010;25(4): 841-848. | C | B | C |
| 27. | Lambrinoudaki I, Kaparos G, Armeni E, Alexandrou A, Damaskos C, Logothetis E, et al. BsmI vitamin D receptor's polymorphism and bone mineral density in men and premenopausal women on long-term antiepileptic therapy. European Journal of Neurology. 2011;18(1):93-8. | C | B | C |
| 28. | Ozel L, Ata P, Ozel MS, Toros AB, Kara M, Unal E, et al. Risk factors for osteoporosis after renal transplantation and effect of vitamin D receptor Bsm I polymorphism. Transplantation Proceedings. 2011;43(3):858-62. | C | C | C |
| 29. | Lambrinoudaki I, Patikas E, Kaparos G, Armeni E, Rizos D, Thoda P, et al. Vitamin D receptor Bsm1 polymorphism, calcium metabolism and bone mineral density in patients with multiple sclerosis: a pilot study. Neurological sciences : official journal of the Italian Neurological Society and of the Italian Society of Clinical Neurophysiology. 2013;34(8):1433-9. Epub 2012/12/06. | C | B | C |
| 30. | El-Shehaby AM, El-Khatib MM, Marzouk S, Battah AA. Relationship of BsmI polymorphism of vitamin D receptor gene with left ventricular hypertrophy and atherosclerosis in hemodialysis patients. Scandinavian Journal of Clinical & Laboratory Investigation. 2013;73(1):75-81. | B | C | C |
| 31. | Phabphal K, Geater A, Limapichat K, Sathirapanya P, Setthawatcharawanich S, Leelawattana R. The association between CYP 2C9 polymorphism and bone health. Seizure. 2013;22(9):766-71. | C | B | C |
| 32. | Schuch NJ, Garcia VC, Vivolo S, Martini LA. Relationship between Vitamin D Receptor gene polymorphisms and the components of metabolic syndrome. Nutrition Journal. 2013;12. | B | C | C |
| 33. | Ghorbanihaghjo A, Argani H, Samadi N, Valizadeh S, Halajzadeh J, Yousefi B, et al. Relationship Between Vitamin D Receptor Gene Fokl and Apal Polymorphisms and Serum Levels of Fetuin-A, Vitamin D, and Parathyroid Hormone in Patients on Hemodialysis. Iranian journal of Kidney Diseases. 2014;8(5):394-400. | C | C | C |
| 34. | Pourfarzam M, Nia KM, Atapour A, Sadeghi HMM. The influence of BsmI and TaqI vitamin D receptor gene polymorphisms on the intensity of hyperparathyroidism in Iranian hemodialysis patients. Advanced Biomedical Research. 2014;3:213. | C | B | C |
| 35. | Santoro D, Gagliostro G, Alibrandi A, Ientile R, Bellinghieri G, Savica V, et al. Vitamin D Receptor Gene Polymorphism and Left Ventricular Hypertrophy in Chronic Kidney Disease. Nutrients. 2014;6(3):1029-37. | B | C | C |
| 36. | Alexandrou A, Armeni E, Kaparos G, Rizos D, Tsoka E, Deligeoroglou E, et al. Bsm1 vitamin D receptor polymorphism and calcium homeostasis following bariatric surgery. Journal of Investigative Surgery. 2015;28(1):8-17. | C | B | C |
| 37. | Grzegorzewska AE, Ostromecki G, Mostowska A, Sowinska A, Jagodzinski PP. Clinical aspects of vitamin D-binding protein gene polymorphisms in hemodialysis patients. Polskie Archiwum Medycyny Wewnetrznej-Polish Archives of Internal Medicine. 2015;125(1-2):8-+. | A | A | C |
| 38. | Oddsson A, Sulem P, Helgason H, Edvardsson VO, Thorleifsson G, Sveinbjörnsson G, et al. Common and rare variants associated with kidney stones and biochemical traits. Nature communications. 2015;6. | A | A | B |
| 39. | Piedra M, Berja A, Garcia-Unzueta MT, Ramos L, Valero C, Amado JA. Rs219780 SNP of Claudin 14 Gene is not Related to Clinical Expression in Primary Hyperparathyroidism. Clinical Laboratory. 2015;61(9):1197-203. | B | B | C |
| 40. | Diaz-Soto G, Romero E, Castrillon JLP, Jauregui OI, Roman DD. Clinical Expression of Calcium Sensing Receptor Polymorphism (A986S) in Normocalcemic and Asymptomatic Hyperparathyroidism. Hormone and Metabolic Research. 2016;48(3):163-8. | C | B | C |
| 41. | Diaz-Soto G, Romero E, Perez-Castrillon JL, Jauregui OI, Roman DD. Parathyroid Hormone Polymorphism RS6254 is Associated with the Development and Severity of Osteoporosis in Asymptomatic but not Normocalcemic Hyperparathyroidism. Hormone and Metabolic Research. 2016;48(12):828-33. | B | A | C |
| 42. | Marchelek-Mysliwiec M, Rozanski J, Ogrodowczyk A, Dutkiewicz G, Dolegowska B, Salata D, et al. The association of the Klotho polymorphism rs9536314 with parameters of calcium-phosphate metabolism in patients on long-term hemodialysis. Renal Failure. 2016;38(5):776-80. | B | C | C |
| 43. | Palmer ND, Divers J, Lu LY, Register TC, Carr JJ, Hicks PJ, et al. Admixture mapping of serum vitamin D and parathyroid hormone concentrations in the African American-Diabetes Heart Study. Bone. 2016;87:71-7. | B | A | C |
| 44. | Wang LY, Zhang P, Wang HF, Qin ZW, Wei KB, Lv XA. Association of vitamin D receptor gene polymorphisms with end-stage renal disease and the development of high-turnover renal osteodystrophy in a Chinese population. Genetics & Molecular Research. 2016;15(2). | A | B | C |
